# Editorial: Biomacromolecules Systems Applied to Medical Implants for the Release of Therapeutic Agents

**DOI:** 10.3389/fbioe.2022.910203

**Published:** 2022-04-27

**Authors:** Joao Henrique Lopes, Nicolas Tabary, Jacobo Hernandez-Montelongo

**Affiliations:** ^1^ Department of Chemistry, Division of Fundamental Sciences (IEF), Aeronautics Institute of Technology - ITA, Sao Jose dos Campos, Brazil; ^2^ Université Lille 1, Unité Matériaux et Transformations (UMET), UMR CNRS 8207, Villeneuve d’Ascq, France; ^3^ Department of Physical and Mathematical Sciences, Catholic University of Temuco, Temuco, Chile

**Keywords:** biomacromolecules, medical implants, controlled release, therapeutic agents, drug release

Medical implants are devices fabricated to replace, aid, or enhance some missing biological structure (Singh et al., 2020). These devices include catheters, stents, hip joints, intraocular lenses, and others. The implant surfaces are designed with biocompatible materials since they will be in direct contact with physiological fluids and tissues, such as titanium, silicone, bioactive glass, polymers, among others. In addition, the choice of the appropriate biomaterial is guided by the presence of desirable properties according to the claimed application (Ratner and Zhang, 2020). Surgery is the most common way to incorporate medical implants into the body, which involves inherent risks: bacterial infections, local swelling and induration, inadequate healing, triggering immune reactions, and other different possible complications (Major et al., 2015). The eventuality and intensity of these postoperative risks are higher in cases of immunosuppression caused by certain diseases or conditions, such as AIDS, cancer, diabetes, malnutrition, and certain genetic disorders (Madsen et al., 2021). In that sense, biomacromolecules are versatile to be configurated as release systems of therapeutic agents to health or significantly reduce those postoperative risks.

This Research Topic collection covers the use of biomacromolecules systems applied to biomedical implants for the release of therapeutic agents. This collection of papers is formed of seven original research articles and one review article.

Different interesting antibiotic drug delivery systems based on biomacromolecules were presented in this Research Topic. New proposes were physiochemically detailed, their biocompatibility was confirmed and their antibacterial activity was tested against both Gram-negative and Gram-positive bacteria. Mansouri et al. obtained an optimal niosomal formulation that exhibited controlled drug release for the antibiotic streptomycin sulfate, with the highest encapsulation efficiency but a minimum size and low polydispersity index. Sun et al. explored a novel temperature-sensitive hydrogel-based on a dual sustained-release system prepared by mesoporous silica (SBA-15) loaded with the antibiotic vancomycin hydrochloride (Van). These Van/SBA-15 particles were encapsulated in a chitosan-glycerophosphate-sodium alginate hydrogel (CS-GP-SA) showing the controlled release of VAN for the treatment of infectious jaw defects. In the same field, Wiggers et al. obtained chitosan films crosslinked with quercetin for the controlled delivery of the antibiotic trimethoprim. Their results showed that quercetin increased the crosslinking degree in a dose-dependent manner, thus influencing the release kinetic of the loaded antibiotic while maintaining its bactericidal effects.

Boas and Reches reported the development of transparent antibacterial and antiviral coatings. This work presented a novel short peptide (NH_2_-DOPA-(Phe)_2_-(His)_6_-OH) that can self-assemble and bind different metal Cu ions. These coatings released monovalent cuprous ions and hydrogen peroxide, providing the surfaces with significant antibacterial effects and antiviral properties.

Antifungal systems were also approached. Pimentel et al. evaluated the antifungal activity against *C. albicans* of silver-containing microcrystals such as silver vanadate (α-AgVO_3_), silver tungstate (α-Ag_2_WO_4_), and silver molybdate (β-Ag_2_MoO_4_). The biocompatibility was tested by an interestingly new three-dimensional coculture model involving three cell lines. Based on the biocompatibility and antifungal findings, α-Ag_2_WO_4_ was the most promising material for treating *C. albicans* infection.

Layer-by-layer (LbL) assembly is a very versatile and significantly inexpensive approach to forming thin films. In this field, Miranda et al. assembled by LbL an extracellular matrix protein fibronectin, as an anionic polymer, with poly-L-lysine (PLL), as a cationic polymer. The fibronectin component was able to create a biomimetic interface, which enhanced the cell attachment of fibroblasts. In addition, the presence of PLL conferred to the films remarkable antibacterial properties against Gram-positive strains.

Cell-based drug delivery has been highlighted as a system to facilitate the transfer of substances to specific organs and tissues with elevated efficiency. In that sense, Litvinova et al. demonstrated that hMSCs can be successfully impregnated with synthetic CaCO_3_ microcapsules without damaging cell structural integrity and mobility. This study established the possibility of using hMSCs as a carrier for a targeted drug delivery system.

Finally, Picker et al. contributed with a review on the use of prokaryotic collagen-like proteins to both study the biology of conventional collagen and develop a new biomaterial platform. Moreover, the authors described the application of Scl2 protein in thromboresistant coating for cardiovascular devices, scaffolds for bone regeneration, chronic wound dressing, and matrices for cartilage regeneration.

In conclusion, this Research Topic provides an overview of recent advances in biomacromolecules systems used for the release of different kinds of therapeutic agents. Moreover, the exploration of the use of innovative carriers transfers substances such as cells and proteins.
